# *Sox2* Expression Is Regulated by a Negative Feedback Loop in Embryonic Stem Cells That Involves AKT Signaling and FoxO1

**DOI:** 10.1371/journal.pone.0076345

**Published:** 2013-10-08

**Authors:** Briana D. Ormsbee Golden, Erin L. Wuebben, Angie Rizzino

**Affiliations:** Eppley Institute for Research in Cancer and Allied Diseases, University of Nebraska Medical Center, Omaha, Nebraska, United States of America; Kanazawa University, Japan

## Abstract

The self-renewal and pluripotency of embryonic stem cells (ESC) is regulated by a highly integrated network of essential transcription factors, which includes Sox2. Previous studies have shown that elevating Sox2 on its own in mouse ESC induces differentiation and inhibits the expression of endogenous Sox2 at the protein and mRNA level. These findings led us to hypothesize that increases in Sox2 activate a negative feedback loop that inhibits the transcription of the endogenous *Sox*2 gene. To test this hypothesis, we used i-OSKM-ESC, which elevate Sox2 in conjunction with Oct4, Klf4, and c-Myc when treated with doxycycline (Dox). Elevating the expression of these four transcription factors in i-OSKM-ESC does not induce differentiation, but it represses expression of endogenous Sox2. We determined that increases of Sox2 in i-OSKM-ESC lead to increases in activated AKT and inactivation of FoxO1 (an activator of *Sox2*), as well as decreases in binding of FoxO1 to the 5'flanking region of *Sox2*. Importantly, we determined that inhibition of AKT in Dox-treated i-OSKM-ESC leads to re-expression of endogenous *Sox2* at the mRNA and protein level and reactivation of FoxO1. These findings argue that AKT signaling is part of the negative feedback loop that helps carefully control the transcription of *Sox2* in ESC by modulating the binding of FoxO1 to the *Sox2* gene. Collectively, our findings provide new insights into the mechanisms that enable ESC to carefully regulate the levels of Sox2 and retain their stem cell properties.

## Introduction

The self-renewal and pluripotency of embryonic stem cells (ESC) is dependent on an intricate network of signaling proteins and transcription factors [1]. Several transcription factors involved in the regulation of ESC, especially Sox2 and Oct4, require tight regulation of their own expression to maintain ESC in a pluripotent stem cell state [2]–[4]. Sox2 and Oct4 are believed to help promote their own transcription by their cooperative binding to adjacent DNA sites in the regulatory regions of each of their genes [3], [5], [6]. Subsequently, it was determined that elevating the level of Sox2 or Oct4 in ESC leads to decreases in the activities of their own promoters [6], [7], and triggers the differentiation of ESC [2], [4]. More recently, it has been determined that Nanog represses the transcription of its own gene [8], [9]. Collectively, these studies indicate that the levels of key pluripotency transcription factors, such as Sox2, Oct4 and Nanog, are carefully regulated in ESC. However, the mechanisms used by ESC to regulate the levels of these transcription factors, in particular Sox2, are poorly understood.

PI3K/AKT/GSK3-β and MEK/ERK signaling have been shown to control the self-renewal and differentiation of ESC [10]–[13]. ESC self-renew and remain pluripotent in serum-free medium without LIF when inhibitors of GSK3-β, MEK and fibroblast growth factor receptors (FGFR) are added to their culture medium [13]. Moreover, increased AKT activity enables ESC to self-renew in serum-containing medium without the addition of LIF [14], which may be due, in part, to the prominent role of AKT signaling in the inhibition of GSK3-β by phosphorylation at serine 9 (S9) [15]. However, AKT has other important roles in pluripotent stem cells. AKT has been reported to phosphorylate essential transcription factors in mouse and human ESC, and in human embryonal carcinoma cells. Oct4 is phosphorylated in human ESC on at least 14 different residues [16]. At least one of these residues, threonine 235 (T235), can be phosphorylated by AKT, which influences Oct4 stability, nuclear accumulation, and transcriptional activity [17]. Other studies have shown that AKT can phosphorylate Sox2 on threonine 118 (T118), which enhances Sox2 stability and increases the ability of Sox2 to reprogram embryonic fibroblasts to induced pluripotent stem cells [18]. Furthermore, it was demonstrated recently that FoxO1 is required for the self-renewal of both human and mouse ESC [19]. Interestingly, this study also reported that FoxO1, which is a target of AKT signaling in other systems [20], is able to bind the conserved regulatory regions upstream of the human *Sox2* gene. Together, these studies raise the possibility that AKT may carefully regulate Sox2 expression in ESC by more than one mechanism. Moreover, they illustrate the critical connections between key signaling pathways and the transcriptional circuitry of ESC [21].

Thus far, the molecular mechanisms and pathways used to “sense” and respond to elevated levels of Sox2 have not been determined. Given the importance of tightly regulating the levels of Sox2 in ESC [4], as well as neural stem cells [22], it is important to elucidate the mechanisms that control the levels of this master regulator. Previously, we determined that elevating Sox2 on its own from a Dox-inducible transgene in ESC (i-Sox2-ESC) leads to rapid decreases in endogenous *Sox2* gene expression as early as 9 hours after Dox treatment, which is only 3 hours after exogenous Sox2 levels are detectable by western blot analysis [4]. The rapid decrease in *Sox2* mRNA levels led us to hypothesize that *Sox2* expression is carefully regulated by an essential signaling network in ESC, which activates a Sox2 negative feedback loop when Sox2 levels become excessive. Recently, we determined that elevating Sox2 in conjunction with Oct4, Klf4 and c-Myc from a Dox-inducible transgene in ESC (i-OSKM-ESC), does not induce differentiation, but represses expression of enodogenous *Sox2* at both the RNA and the protein level [23]. To test our hypothesis and begin to understand the mechanisms involved, we examined whether key signaling factors control the expression of the endogenous *Sox2* gene when the protein levels of Sox2 rise in ESC.

## Materials and Methods

### Cell Culture Conditions

Stock cultures and experimental cultures of i-OSKM-ESC were maintained as described previously [23]. For experiments involving transgene induction, 1.5×10^6^ i-OSKM-ESC were cultured in 100 mm dishes with 4 μg/ml Dox for 48 hours unless otherwise indicated. After an initial 24 hours of cell culture in the absence or presence of Dox, some of the i-OSKM-ESC were treated with small molecules for the remaining 24 hours unless otherwise indicated. The small molecules used were dissolved in DMSO and used at the concentrations listed in Table S1. All experimental cultures of i-OSKM-ESC (including an untreated control) were exposed to the same concentration of DMSO for the same amount of time.

### Extract Preparation and Western Blotting

Nuclear and Cytoplasmic protein extracts from untreated and Dox-treated i-Sox2-ESC and i-OSKM-ESC were prepared using the Pierce NE-PER^TM^ nuclear and cytoplasmic extraction kit (Pierce, Thermo Fisher Scientific, Rockford, IL), as described previously [4]. Endogenous Sox2 migrated as an ∼35 kDa protein and Flag-Sox2 migrated at a slower rate due to the highly acidic Flag epitope, as described previously [4]. The presence of alkaline phosphatase conjugated secondary antibodies was detected using the enhanced chemiflourescence kit (Amersham Biosciences, Piscataway, NJ) and scanned on a TyphoonFLA 7000 imager (GE Healthcare, Pittsburgh, PA). To detect multiple proteins on a single membrane, we followed the protocol for the Restore Western Blot Stripping Buffer (Pierce, Thermo Fisher Scientific, Rockford, IL) between each antibody. The antibodies used are listed in Table S2.

### RNA Isolation and cDNA Synthesis

i-OSKM-ESC were seeded in 100 mm dishes at a density of 1.5×10^6^ in the absence or presence of Dox and/or AKTiV, and RNA was isolated as described previously [4]. cDNA synthesis was performed with 0.5 μg of RNA using the AccuScript High Fidelity 1st Strand cDNA Synthesis Kit, Cat # 200820 (Agilent Technologies, Stratagene, La Jolla, CA) for reverse transcription (RT).

### Quantitative Polymerase Chain Reaction (qPCR)

cDNA generated by reverse transcription (RT) using RNA from i-OSKM-ESC cultured in the absence or presence of Dox and/or AKTiV were analyzed using RT^2^ SYBR® Green qPCR Mastermix Cat # 330502 (Qiagen, Germantown, MD) for qPCR. The qPCR reactions were prepared in triplicate in 96-well Blu/Wht Hard-shell PCR Plates Cat # HSP9635 (BioRad, Hercules, CA), plates were covered with Microseal ‘B’ Film Cat # MSB1001 (BioRad, Hercules, CA), and qPCR was carried out in the CFX96 Real-TimeSystem with a C100 Thermal Cycler (BioRad, Hercules, CA). Previously described gene-specific primers for *Sox2* 3'UTR [4] and *GAPDH* [24] were used in qPCR analysis of cDNA. Relative gene expression for each gene in the untreated and Dox-treated ESC was normalized to GAPDH. Gene expression for Dox-treated cells is reported as the average fold change relative to the expression of the gene in the untreated cells.

### TaqMan RT and TaqMan qPCR for MicroRNA Assay

RT of RNA isolated from i-OSKM-ESC cultured in the absence or presence of Dox was conducted following the protocol provided by Applied Biosystems for TaqMan MicroRNA Assays using the TaqMan® MicroRNA Reverse Transcription Kit and the 5X miRNA RT primers for RNU6B (Assay ID –001093), hsa-miR-145 (Assay ID –002278), hsa-miR-296-5p (Assay ID –000527), hsa-miR-134 (Assay ID –001186), and hsa-miR-21 (Assay ID –000397) (Applied Biosystems, Carlsbad, CA). qPCR of the RT product was conducted following the protocol provided by Applied Biosystems using the TaqMan® Universal PCR Master Mix 2×, No AmpErase® UNG (Applied Biosystems, Carlsbad, CA) and the TaqMan MicroRNA Assay (20X) probes for the primers listed above. qPCR was performed in triplicate using the 96-well Hard-shell PCR Plates and microseals described above. Reactions were carried out in the CFX96 Real-Time System with a C100 Thermal Cycler (BioRad, Hercules, CA) using conditions suggested by the Applied Biosystems protocol.

### mRNA Stability

i-OSKM-ESC were seeded in 100 mm dishes at a density of 1.5×10^6^ in the absence or presence of Dox for 48 hours and/or AKTiV for 24 hours. Prior to RNA isolation, cells were treated with 5 µg/ml actinomycin D for 0, 45, 90, and 180 minutes. RNA was then isolated from the cells, and cDNA synthesis and qPCR analysis was performed as described above.

### Chromatin Immunoprecipitation (ChIP)

ChIP was performed as described previously [25], utilizing 2×10^6^ i-OSKM-ESC per 100 mm plate that were plated in the absence or presence of 4 µg/ml Dox and grown for 24 hours. The cells were refed with or without 4 µg/ml Dox for an additional 24 hours and then treated with formaldehyde to cross-link proteins to DNA, as described previously [25], except for an extension of the formaldehyde incubation and wash times to 10 minutes instead of 5 minutes. The chromatin was sheared to a length of ∼500–700 bp by sonication using a Bioruptor (Diagenode, New York, NY) with sonication cycles consisting of 30 seconds “on” and 30 seconds “off” for five minutes and five minute incubations of tubes on ice between each round for three rounds of five-minute sonications. Sonicated DNA was diluted with ChIP dilution buffer and one percent of the sheared chromatin was removed prior to immunoprecipitation to serve as input DNA in our analysis. Overnight immunoprecipitation was carried out using the remaining chromatin by incubating with 6 µg of the FoxO1 antibody (N-18) sc-9809 from Santa Cruz, 1∶1000 of the FoxO1 antibody CST #2880 from Cell Signaling Technology, or 3 µg of IgG control (GFP antibody SC-9996 from Santa Cruz). Immune complexes were collected by incubating with 60 µl of protein G agarose/salmon sperm DNA bead slurry (Upstate, Lake Placid, NY) for one hour at 4°C.

### Quantitative polymerase chain reaction (qPCR) for ChIP DNA

For ChIP analysis, enrichment at each FoxO1 binding element (FBE) within the 5' flanking region of *Sox2* was compared to a control region located ∼4 kb downstream of the *Sox2* transcription start site, which contains the adjacent binding sites of Sox2 and Oct4 [6], [26]. Enrichment of immunoprecipitated DNA was determined using RT^2^ SYBR® Green qPCR Mastermix Cat # 330502 (Qiagen, Germantown, MD), as described above, using the primer sequences provided in Table S3. Primer curves were calculated to determine fold changes (Table S3). Fold change of FoxO1 enrichment at a specific region relative to the control region was calculated by comparing the normalized Ct values (normalized to input) for each region in triplicate. This fold change of FoxO1 enrichment was determined using two different FoxO1 antibodies (listed above). The fold change of IgG immunoprecipitation of each FBE site and the control region was calculated as described above, and the fold change of each FoxO1 antibody enrichment was normalized to the IgG enrichment before obtaining an average of the triplicate results and determining the standard error of the mean.

## Results

### Elevated levels of exogenous Sox2 lead to decreases in endogenous Sox2 expression and increases in phosphorylated AKT

**Figure 1:**
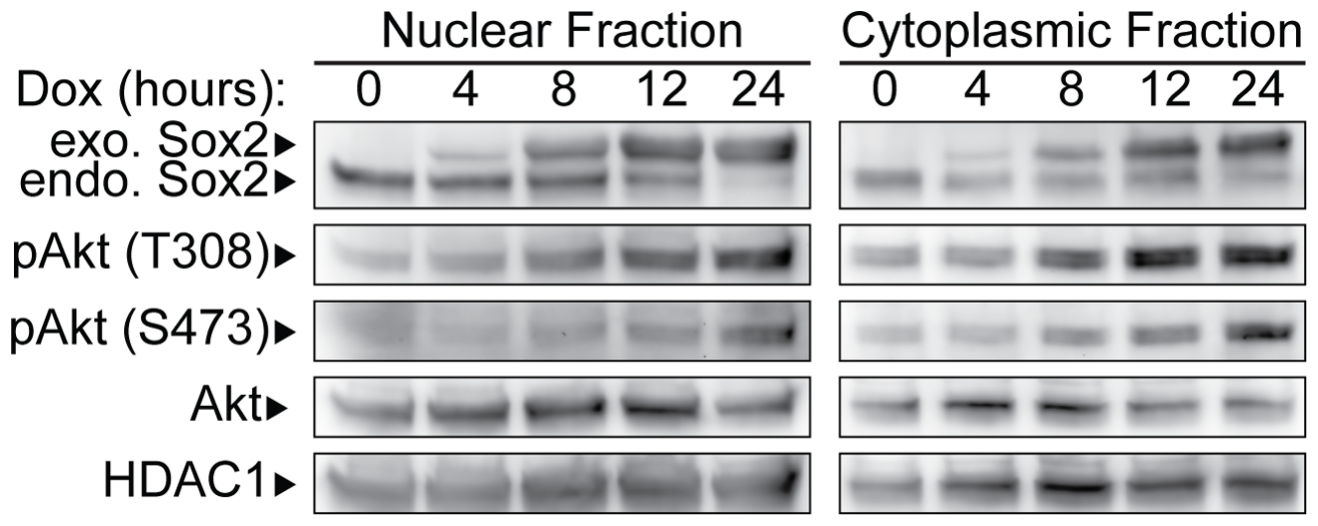
Time course of endogenous Sox2 inhibition. i-OSKM-ESC were seeded at 1.5×10^6^ per 100 mm dish and cultured for 24 hours. The cells were refed with fresh medium with or without 4 μg/ml Dox for the number of hours indicated and nuclear and cytoplasmic extracts were prepared from the cells. Equal amounts of nuclear and cytoplasmic protein were loaded into each well of an SDS-PAGE and western blot analysis was performed by sequentially probing for Sox2, pAKT(T308), and pAKT(S473), total AKT and HDAC1. HDAC1 served as a protein loading control.

To gain a better understanding of the mechanisms that regulate Sox2 expression in ESC, we initially examined the phosphorylation status of AKT after elevating the levels of Sox2 in i-OSKM-ESC, because AKT signaling, and thereby inhibition of GSK3-β, is essential for the self-renewal and pluripotency of ESC [12], [14]. More specifically, we examined whether rapid decreases of endogenous Sox2 protein levels in i-OSKM-ESC upon elevation of exogenous Sox2 with Dox treatment was accompanied by changes in AKT signaling. We initially examined whether phosphorylated AKT levels correlate with the increases of Sox2 expression in Dox-treated i-OSKM-ESC. For these studies, nuclear and cytoplasmic protein extracts were prepared from i-OSKM-ESC cultured in the absence and presence of Dox for 4, 8, 12, and 24 hours (Figure 1). In these cells, exogenous Sox2 expression was detectable at 4 hours of Dox treatment and was significantly elevated by 8 hours, and exogenous Sox2 continued to increase through 24 hours of Dox treatment (Figure 1). Interestingly, endogenous Sox2 begins to decrease after 12 hours of Dox treatment and its expression is dramatically reduced after 24 hours of Dox treatment (Figure 1). Nuclear and cytoplasmic protein fractions were also subjected to western blot analysis and probed with antibodies that recognize the phosphorylation of AKT at residue threonine 308 [pAKT(T308)], which can be phosphorylated by PDK1 [27], and at residue serine 473 [pAKT(S473)], which can be phosphorylated by mTORC2 [28]. These two residues of AKT are phosphorylated independently [29] and phosphorylation of AKT at one or both of these residues is important for AKT activity [30]. An increase in pAKT(T308) was first detected within 8 hours of Dox treatment, and remained elevated over the next 16 hours (Figure 1). Increases in pAKT(S473) were also observed, but they increased more slowly than increases in pAKT(T308). Thus, increases of pAKT(T308) parallel closely with increases in exogenous Sox2 expression; whereas, decreases of endogenous Sox2 expression parallel more closely with increases in pAKT(S473).

### Endogenous Sox2 is re-expressed in response to AKT inhibition

**Figure 2:**
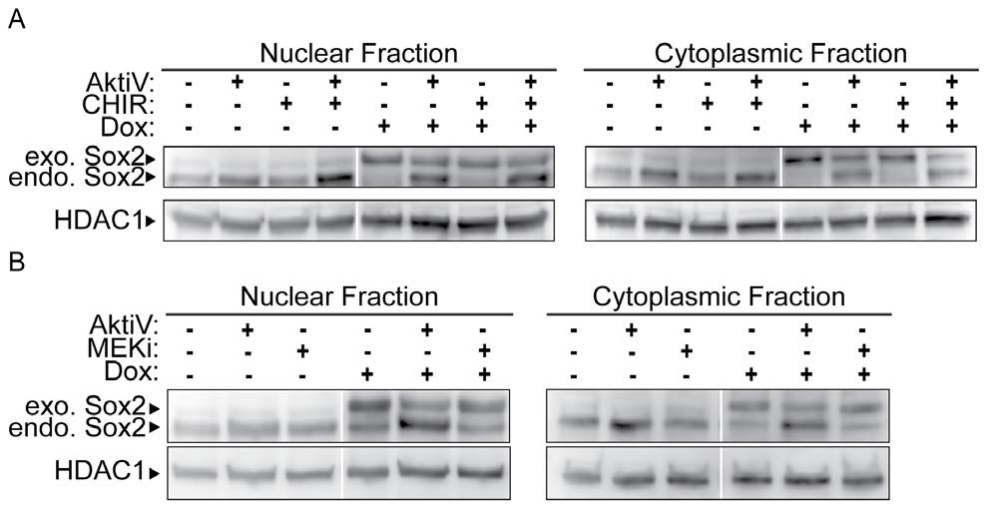
Effects of AKT, GSK3 and MEK inhibitors on the expression of endogenous Sox2. 1.5×10^6^ i-OSKM-ESCs were cultured in 100 mm dishes with or without 4 μg/ml Dox. (A) 24 hours after the cells were plated in the absence or presence of Dox, 5 µM AKTiV and 3 µM CHIR were added to the cells for an additional 24 hours where indicated. (B) 24 hours after the cells were plated in the absence or presence of Dox, 5 µM AKTiV and 0.4 µM MEKi were added to the cells for an additional 24 hours where indicated. For A-B, nuclear and cytoplasmic protein extracts were prepared from the cells and equal amounts of nuclear and cytoplasmic protein were loaded into each well of an SDS-PAGE. Western blot analysis was performed by sequentially probing for Sox2 and HDAC1 in A and B. In each case, HDAC1 served as a protein loading control.

To determine whether increases in AKT activation affect the endogenous expression of Sox2, we cultured Dox-treated i-OSKM-ESC with inhibitors that block the activation of AKT as well as inhibitors that block the activity of other signaling proteins whose regulation is important for sustaining the self-renewal of ESC. Initially, we treated i-OSKM-ESC with the AKT inhibitor, Triciribine (AKTiV), the GSK3-β inhibitor, CHIR99021 (CHIR; which may also inhibit GSK3-α), or the MEK inhibitor, PD0325901 (MEKi). As expected from our findings discussed above, western blot analysis of both nuclear and cytoplasmic protein fractions determined that i-OSKM-ESC treated with Dox for 48 hours exhibited elevated levels of exogenous Sox2 and close to a complete shut-off of endogenous Sox2 protein (Figure 2A). Importantly, when Dox-treated i-OSKM-ESC were cultured with AKTiV, expression of endogenous Sox2 was no longer blocked (Figure 2A). In contrast, inhibition of GSK3-β with CHIR did not restore the activation of endogenous Sox2 expression when Sox2 is elevated (Figure 2A). Moreover, simultaneous inhibition of GSK3-β and AKT did not block the re-expression of endogenous Sox2 observed with AKT inhibition on its own in the presence of Dox. Similar to inhibition of GSK3-β, inhibition of MEK signaling alone, or in combination with Dox treatment, did not influence the endogenous Sox2 or exogenous Sox2 expression (Figure 2B). In addition, neither the AKT inhibitor, the GSK3-β inhibitor nor the MEK inhibitor on their own significantly influenced the expression of Sox2 protein in the control i-OSKM-ESC that were not treated with Dox (Figure 2). Together, these studies argue that AKT regulates the expression of endogenous Sox2 when Sox2 levels are elevated in ESC. In this connection, expression of the endogenous Oct4 and Klf4, which are not affected when i-OSKM-ESC are treated with Dox, exhibit little or no change when these cells were treated with AKTiV in the presence or absence of Dox (data not shown).

**Figure 3:**
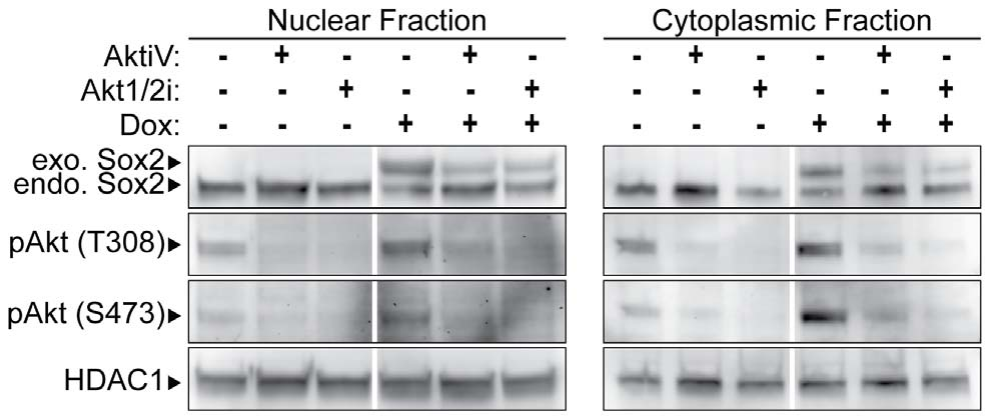
Effects of two different AKT inhibitors on the expression of endogenous Sox2. i-OSKM-ESC were seeded at 1.5×10^6^ per 100 mm dish with or without 4 μg/ml Dox for 24 hours. (A) After the initial 24 hours, the cells were refed with fresh medium with or without 4 μg/ml Dox, and treated with 5 μM AKTiV or 5 μM AKT1/2i for an additional 24 hours where indicated. 48 hours after the cells were plated, nuclear and cytoplasmic protein extracts were prepared and equal amounts of nuclear and cytoplasmic protein were loaded into each well of an SDS-PAGE. Western blot analysis was performed by sequentially probing for pAKT(T308), pAKT (S473), Sox2 and HDAC1, which served as a protein loading control.

To examine whether the effects of AKT inhibition are specific to the AKT pathway and not an off-target effect of AKTiV, we tested a second AKT inhibitor, referred to here as AKT1/2i. Inhibition of AKT with AKT1/2i led to the re-expression of endogenous Sox2, similar to that observed with AKTiV (Figure 3). Thus, expression of endogenous Sox2 appears to be specifically regulated by AKT signaling. We also examined the phosphorylation status of AKT in i-OSKM-ESC cultured with each AKT inhibitor, and, as expected, we observed a decrease in the phosphorylation of AKT at T308 and S473 (Figure 3). Interestingly, inhibition of AKT with each AKT inhibitor in combination with Dox led to decreases in exogenous Sox2 protein (Figure 3). The decrease in exogenous Sox2 protein after inhibition of AKT can be explained by previous reports that AKT can enhance the stability of Sox2 protein by phosphorylation at threonine 118 (T118) [18]. We also detected small increases in the levels of Sox2 primarily in the cytoplasmic fraction when the cells were treated with an AKT inhibitor without Dox (Figure 2A); however, the small increase in Sox2 was not consistently observed. It is likely that this increase in Sox2 protein expression following AKT inhibition in the absence of Dox is difficult to detect, because AKT regulates Sox2 in two opposing ways: it phosphorylates Sox2 on T118, which promotes Sox2 stability [18], but it also reduces endogenous Sox2 expression at the RNA level (see below). Thus, the regulation of Sox2 by AKT signaling in ESC is highly sensitive and multifaceted. This point is addressed more fully in the Discussion section. Collectively, our studies argue that the elevation of Sox2 in ESC specifically activates a Sox2 negative feedback loop that is mediated, at least in part, by AKT signaling.

To determine whether other signaling pathways upstream of AKT influence endogenous Sox2 expression when i-OSKM-ESC were treated with Dox, we tested the effects of inhibitors that block the activities of FGFR, phosphatase and tensin homolog (PTEN), and Src (Figure S1). Inhibition of FGFR in Dox-treated i-OSKM-ESC did not restore the expression of endogenous Sox2 (Figure S1A). In fact, this inhibitor reduced the expression of exogenous Sox2 levels, possibly due to destabilizing Sox2 at the protein level. Similarly, inhibition of Src and PTEN did not restore endogenous Sox2 levels to those seen in the untreated cells (Figure S1B,C). Finally, we examined whether treatment of the i-OSKM-ESC with Dox alters the expression of ERas. Recent studies have linked ERas and signaling by AKT [31]. However, we observed little or no change in expression of ERas when Sox2 levels decreased following Dox-treatment of i-OSKM-ESC (Figure S1D).

### PI3K activates AKT, but S6K is not a downstream effecter of the negative feedback loop between Sox2 and AKT

**Figure 4:**
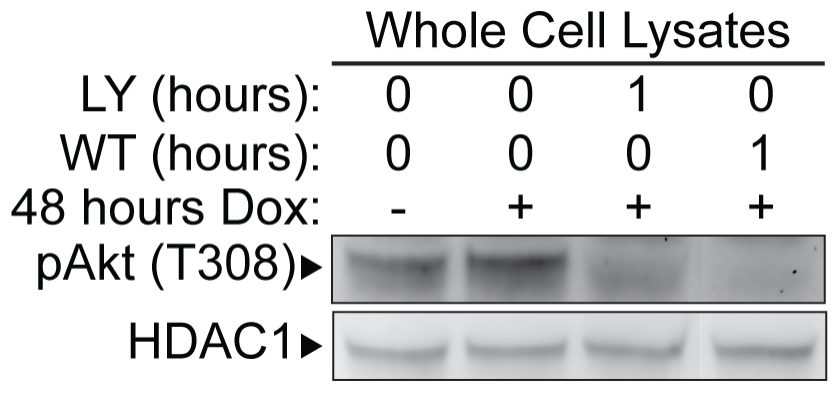
Effects of PI3K inhibitors on the phosphorylation of AKT. i-OSKM-ESC were seeded at 1.5×10^6^ per 100 mm dish with or without 4 μg/ml Dox for 48 hours. Where indicated, the cells were treated during the last hour with either 10 μM LY294002 (LY) or 200 nM Wortmannin (WT). 48 hours after the cells were plated, whole cell extracts were prepared and equal amounts of protein were loaded into each well of an SDS-PAGE. Western blot analysis was performed by sequentially probing for pAKT(T308) and HDAC1, which served as a protein loading control.

An obvious candidate responsible for activation of AKT signaling is PI3K, because PI3K has been shown to activate both PDK1 and mTORC2 [27], [32], which phosphorylate AKT at T308 and S473, respectively [27], [28]. To test whether the inhibition of PI3K signaling would block the increase in AKT phosphorylation observed when i-OSKM-ESC are treated with Dox, Dox-treated i-OSKM-ESC were cultured in the absence and presence of two PI3K inhibitors, LY294002 and Wortmannin. Western blot analysis of whole cell lysates demonstrated that inhibition of PI3K not only blocks enhanced activation of AKT in the presence of Dox, it also leads to decreases in AKT phosphorylation below the level found in untreated i-OSKM-ESC (Figure 4). These findings suggest that the increases in AKT activity following the elevation of exogenous Sox2 in Dox-treated i-OSKM-ESC are dependent on PI3K signaling.

**Figure 5:**
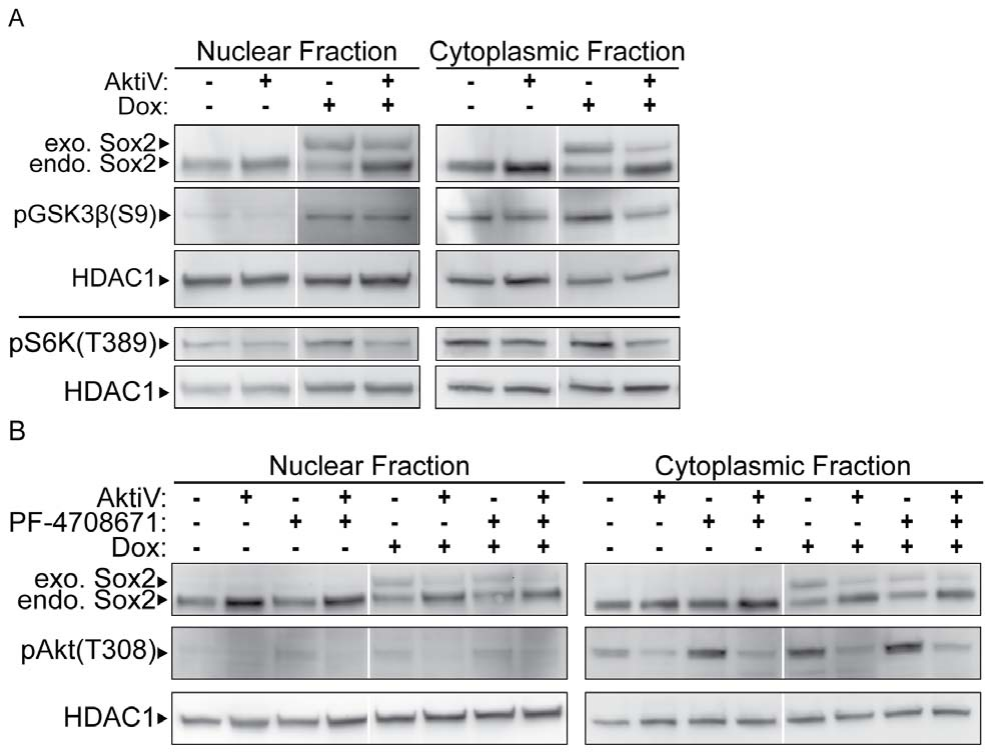
Effects of elevating Sox2 on phosphorylation of GSK3β and S6K. i-OSKM-ESC were seeded at 1.5×10^6^ per 100 mm dish with or without 4 μg/ml Dox for 24 hours. (A) After the initial 24 hours, the cells were refed with fresh medium with or without 4 μg/ml Dox, and treated with 5 μM AKTiV for an additional 24 hours where indicated. 48 hours after the cells were plated, nuclear and cytoplasmic protein extracts were prepared and equal amounts of nuclear and cytoplasmic protein were loaded into each well of an SDS-PAGE. Western blot analysis was performed by sequentially probing for pGSK3β(S9), Sox2 and HDAC1 (top) or pS6K(T389) and HDAC1 (bottom). (B) After the initial 24 hours, the cells were refed with fresh medium with or without 4 μg/ml Dox, and treated with 5 μM AKTiV and/or PF-4708671 for an additional 24 hours where indicated. As in A, nuclear and cytoplasmic extracts were prepared from the cells and western blot analysis was performed by sequentially probing for pAKT(T308), Sox2 and HDAC1. In each case, HDAC1 served as a protein loading control.

To determine which proteins downstream of AKT signaling contribute to the inhibition of endogenous Sox2 expression when exogenous Sox2 levels rise and AKT activation is increased, we examined the phosphorylation status of pGSK3-β(S9) and S6 kinase on threonine 389 [pS6K(T389)]. As described earlier, activated AKT leads to the inhibition of GSK3-β by phosphorylation at S9. Activated AKT also leads to the upregulation of mTORC1 signaling, which phosphorylates S6K on threonine 389, thereby activating S6K [33]. As expected, western blot analysis indicated that both the phosphorylation of pGSK3-β(S9) and pS6K(T389) increased when i-OSKM-ESC were treated with Dox; whereas, the increases in phosphorylation of pGSK3-β(S9) and pS6K(T389) were not observed when an AKT inhibitor was added to the Dox treated cells (Figure 5A).

Our earlier studies (Figure 2A) argue that GSK3-β is not likely to play a significant role in the regulation of endogenous Sox2 expression. To determine whether S6K plays a role in Sox2 regulation, we treated i-OSKM-ESC with the S6K inhibitor, PF-4708671, in the absence and presence of Dox. Western blot analysis of nuclear and cytoplasmic protein extracts demonstrated that inhibition of S6K alone did not increase endogenous Sox2 levels, but it did enhance pAKT(T308) (Figure 5B), likely because inhibition of S6K can release its inhibition of IRS1/PI3K/PDK1 signaling [34], [35]. In addition, when PF-4708671 was combined with AKTiV and Dox, it did not alter the effect of AKT inhibition, which increased endogenous Sox2 expression (Figure 5B). Thus, S6K signaling does not appear to contribute to the Sox2 negative feedback loop. We also examined the effects of rapamycin; however, similar to the effects of PF-4708671 described above, rapamycin elevated the levels of AKT phosphorylation (data not shown) and it was not examined further. As discussed later in this report, we also examined one other AKT target, FoxO1, because it has been shown recently to bind to the human *Sox2* gene [19].

### AKT decreases the expression of the endogenous *Sox2* gene at the RNA level

**Figure 6:**
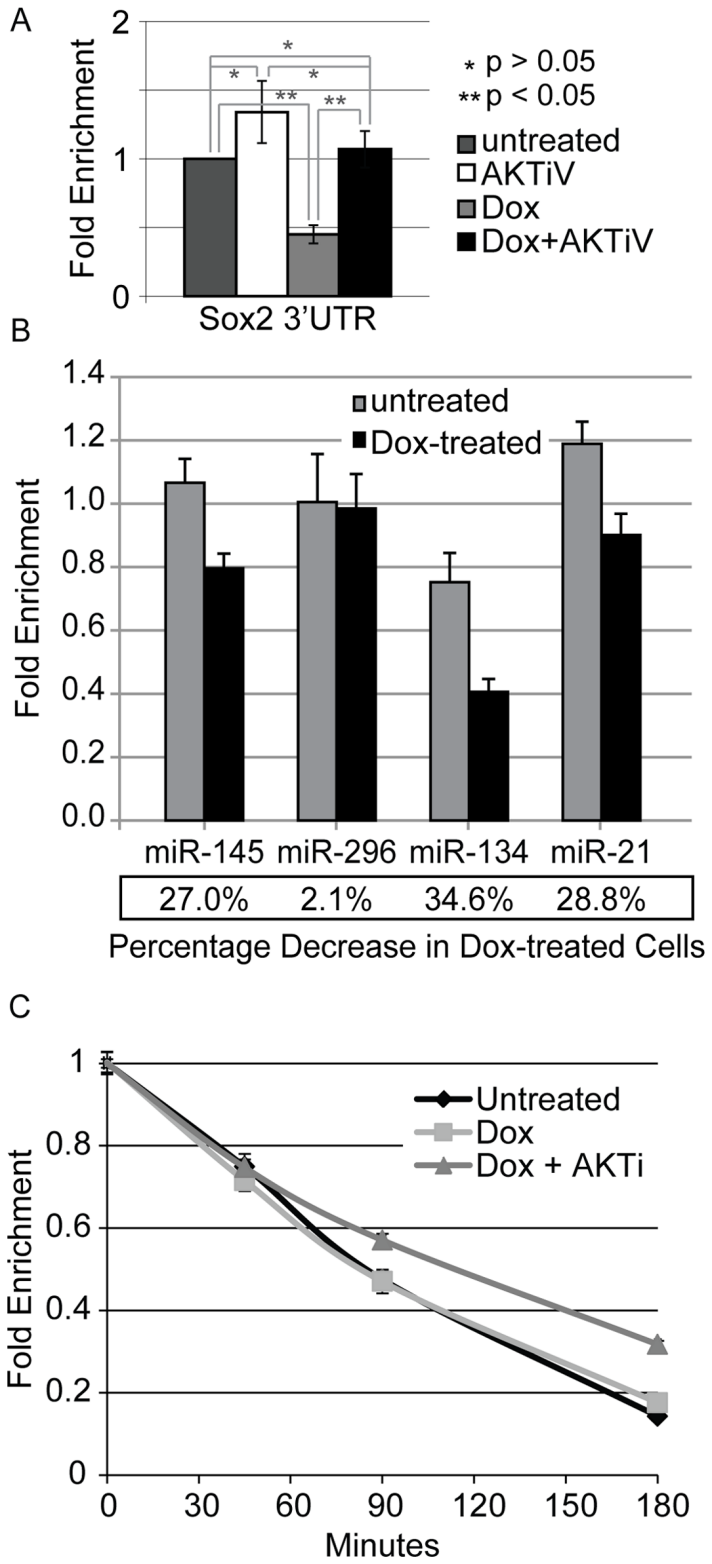
Changes in *Sox2* mRNA and miRNA expression induced by increases in Sox2 protein. i-OSKM-ESC were seeded at 1.5×10^6^ per 100 mm dish with or without 4 μg/ml Dox for 24 hours. (A) After the initial 24 hours, the cells were refed with fresh medium with or without 4 μg/ml Dox and/or 5 μM AKTiV for an additional 24 hours where indicated. 48 hours after the cells were plated, total RNA was isolated from the cells, and RT-qPCR was performed. A primer set specific to the untranslated region at the 3'-end (3'UTR), measuring endogenous Sox2 mRNA, was used in qPCR analysis performed in triplicate. Results are presented as the average fold change of mRNA expression, normalized to GAPDH expression, and compared to untreated i-OSKM-ESC. Statistical significance was determined by Student's t-test and indicated with asterisks: *p>0.05, **p<0.05. Detection of the Dox-inducible transgene expression may be underestimated due to possible limitations of the reverse transcription of the long poly-cistronic transcript that encodes for Oct4, Sox2, Klf4, and c-Myc. (B) After an initial 24 hours, i-OSKM-ESC were refed with fresh medium with or without 4 μg/ml Dox for an additional 24 hours, followed by isolation of total RNA. RT-qPCR using TaqMan probes was performed in triplicate to determine the expression of miR-145, miR-296, miR-134, and miR-21, normalized to RNU6B expression, and reported as average differences in fold change from RNU6B expression. (C) i-OSKM-ESC were seeded at 0.6×10^6^ per 60 mm dish with or without Dox (4 μg/ml). After an initial 24 hours, the cells were refed with fresh medium with or without 4 μg/ml Dox and with 5 μM AKTiV for an additional 24 hours where indicated. RNA synthesis was blocked with 5 µg/ml actinomycin D and total RNA was isolated from the cells at 0, 45, 90, and 180 minutes after actinomycin D treatment (48 hours after plating). RT-qPCR was performed with a primer set specific to the untranslated region at the 3'-end (3'UTR) to measure the remaining endogenous Sox2 mRNA. Results are presented as the average fold change of mRNA expression, normalized to GAPDH expression, and shown as a percentage of remaining mRNA compared to the amount of mRNA present in each treatment before the addition of actinomycin D. Error bars represent standard error of the mean, n = 3. This experiment was repeated with longer periods of actinomycin D treatments and similar results were observed.

Downregulation of the endogenous *Sox2* gene at the RNA level [23] and the upregulation of AKT phosphorylation when i-OSKM-ESC are treated with Dox (Figure 1), led us to test whether inhibition of AKT would increase the expression of the endogenous *Sox2* gene. For these studies, *Sox2* mRNA was monitored by RT-qPCR using a *Sox2* primer set that is specific to the *Sox2* 3'UTR, which detects mRNA encoded by the endogenous *Sox2* gene. After normalizing *Sox2* expression to *GAPDH* mRNA levels, we observed that treatment of i-OSKM-ESC with AKTiV led to a small increase (∼1.4-fold) in *Sox2* mRNA compared to untreated i-OSKM-ESC, but this increase was not statistically significant (Figure 6A). In contrast, expression of the endogenous *Sox2* gene (Sox2 3'UTR) was reduced in the Dox-treated i-OSKM-ESC (∼50% decrease), and this reduction was reversed when Dox treatment was accompanied by inhibition of AKT (Figure 6A). Both the reduction in *Sox2* mRNA when i-OSKM-ESC were treated with Dox and the reversal when AKTiV was added along with Dox were statistically significant (p<0.05).

The reduction in expression of endogenous *Sox2* when i-OSKM-ESC are treated with Dox is likely to result from a decrease in the transcription of the endogenous *Sox2* gene (see below). However, the reduction in endogenous *Sox2* mRNA could be due to changes in post-transcriptional regulation, in particular by miRNAs that have been shown recently to regulate *Sox2* mRNA. To test this possibility, we measured the levels of four miRNAs. The expression of miR-145 was measured, because it is reported to target the 3'UTR of *Sox2* transcripts [36]. The levels of miR-134 and miR-296 were measured, because they are reported to target the coding sequence of *Sox2* mRNA [37]. We also measured the levels of miR-21, because miR-21 expression was reported to correlate with Sox2 expression during embryogenesis [38]. TaqMan miRNA assays indicated that the levels of miR-145, miR-134 and miR-21 decreased when the i-OSKM-ESC were treated with Dox; whereas, miR-296 did not change under these conditions (Figure 6B). Thus, decreases in endogenous *Sox2* mRNA expression when Dox is added to i-OSKM-ESC does not appear to be due to increases in miRNAs known to target *Sox2* mRNA.

To extend these studies, we also examined whether the reduction of endogenous Sox2 when Dox was added to i-OSKM-ESC was due to changes in the stability of *Sox2* mRNA. For these studies, i-OSKM-ESC were cultured in the presence of actinomycin D, which blocks transcription (Figure 6C). We determined that treatment of i-OSKM-ESC with Dox did not alter the turnover of *Sox2* mRNA. In the presence and absence of Dox, *Sox2* mRNA exhibited a half-life of ∼85 minutes. Thus, decreases in endogenous *Sox2* mRNA expression when Dox is added to i-OSKM-ESC does not appear to be due to decreases in the stability of *Sox2* mRNA. Interestingly, the half-life of *Sox2* mRNA increased to ∼115 minutes when AKTiV was added to the Dox-treated i-OSKM-ESC. The potential impact of AKTiV on the half-life of *Sox2* mRNA and the restoration of endogenous Sox2 expression is discussed in the Discussion section.

### Increases in AKT signaling increase phosphorylation of FoxO1, which decreases FoxO1 binding to the 5' regulatory region of *Sox2*

**Figure 7:**
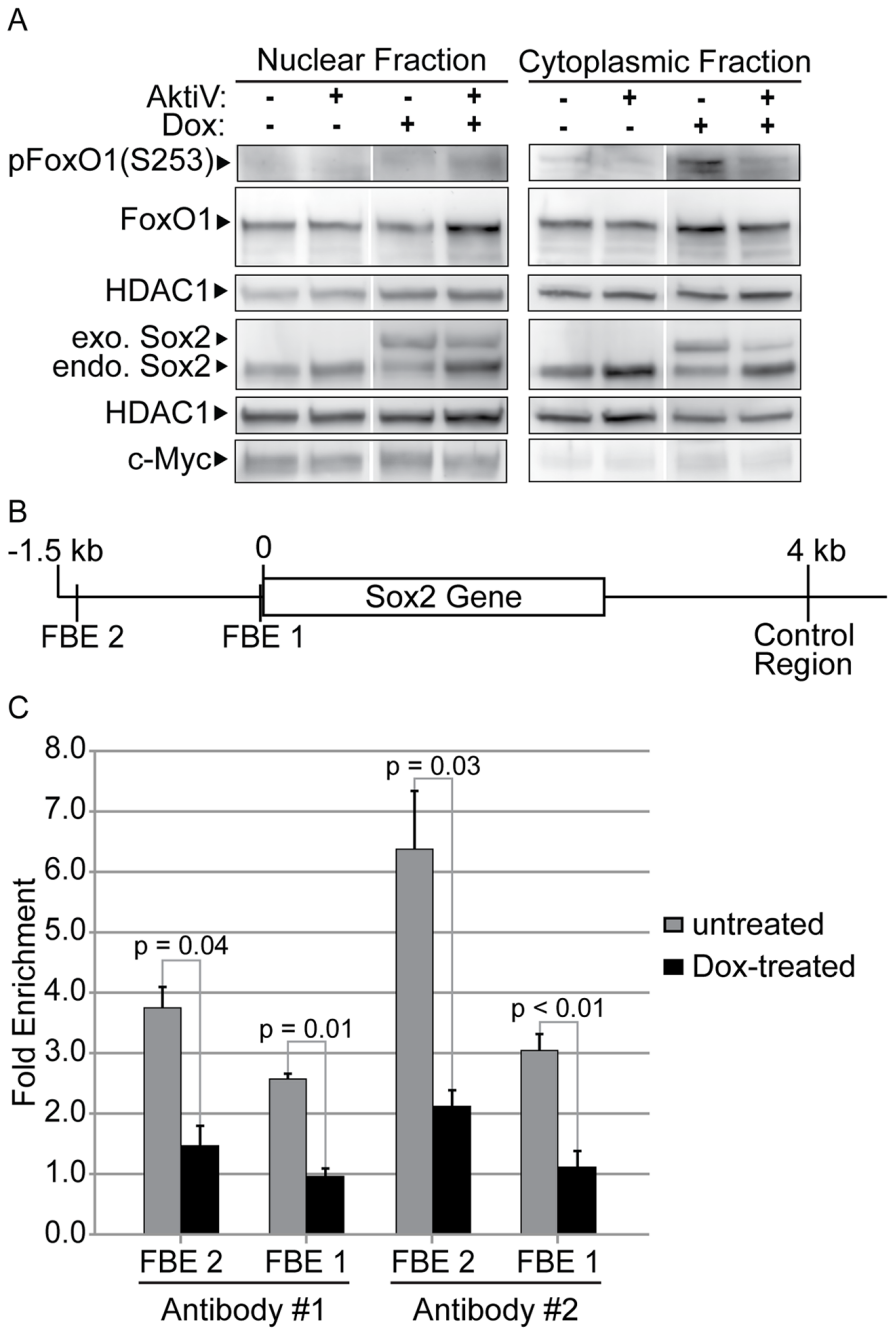
Elevated Sox2 alters FoxO1 phosphorylation and its binding to regulatory regions of the *Sox2* gene. i-OSKM-ESC were seeded at 1.5×10^6^ per 100 mm dish with or without 4 μg/ml Dox for 24 hours. (A) After the initial 24 hours, the cells were refed with fresh medium with or without 4 μg/ml Dox, and treated with 5 μM AKTiV for an additional 24 hours where indicated. 48 hours after the cells were plated, nuclear and cytoplasmic protein extracts were prepared and equal amounts of nuclear and cytoplasmic protein were loaded into each well of an SDS-PAGE. Western blot analysis was performed by sequentially probing for pFoxO1(S253), total FoxO1, Sox2, HDAC1, and c-Myc. HDAC1 was used as a loading control for two separate western blots performed with the same protein extracts. c-Myc was used as a loading control to confirm proper separation of nuclear and cytoplasmic extracts. The levels of c-Myc in nuclear and cytoplasmic extracts were monitored simultaneously in western blots that were imaged together. (B) Schematic of the location of FBE1, FBE2, and the Sox2 control region within the regulatory regions of the *Sox2* gene. (C) Chromatin immunoprecipitation (ChIP) analysis of DNA immunoprecipitated from untreated or Dox-treated i-OSKM-ESC using two different anti-FoxO1 antibodies (#1: Cell Signaling Technology, #2: Santa Cruz) and an IgG (GFP) control antibody for normalization. Analysis was performed in triplicate by qPCR and the values for the FoxO1 immunoprecipitated DNA were normalized to the input samples and the control antibody. The fold change of FoxO1 binding was determined by comparing FoxO1 antibody immunoprecipitation of FBE1 and FBE2 to immunoprecipitation of the Sox2 control region from untreated and Dox-treated i-OSKM-ESC. The results are presented as averages of the triplicate results and error is presented as the standard error of the mean. These findings are statistically significant with p-values all <0.05, as determined by Student's t-test.

Collectively, the findings discussed above suggest that the downregulation of endogenous *Sox2* mRNA when i-OSKM-ESC are treated with Dox is due to an increase in the activity of AKT and a reduction in endogenous *Sox2* transcription. This led us to examine whether AKT influenced the phosphorylation status of FoxO1. Unphosphorylated FoxO1 binds the promoter of the human *Sox2* gene, and it is essential for the pluripotency of ESC [19]. For our studies, we monitored levels of FoxO1 and phosphorylated FoxO1 at serine residue 253 [pFoxO1(S253)] before and after treatment of i-OSKM-ESC with Dox. Western blot analysis demonstrated that pFoxO1(S253) increased in the cytoplasmic protein fraction of Dox-treated i-OSKM-ESC and this increase in phosphorylated FoxO1 was blocked by AKTiV treatment (Figure 7A). Moreover, when phosphorylation of FoxO1 increased, there was a shift in its localization from the nucleus to the cytoplasm. The fractionation of nuclear and cytoplasmic extracts was verified by western blot analysis by monitoring the expression of the transcription factor c-Myc, which was readily detected in the nuclear extracts, but barely detected in the cytoplasmic extracts (Figure 7A). Consistent with the increase in phosphorylated FoxO1 in the cytoplasm, there was a decrease in the level of total FoxO1 protein in the nucleus of Dox-treated i-OSKM-ESC and a corresponding increase in total FoxO1 protein in the cytoplasm (Figure 7A). Furthermore, when i-OSKM-ESC were treated with Dox and AKTiV, FoxO1 phosphorylation decreased and total FoxO1 increased in the nucleus (Figure 7A). Thus, these findings argue that elevation of activated AKT leads to decreases in endogenous Sox2 and increases in phosphorylated FoxO1 in the cytoplasmic compartment.

The increase in FoxO1 phosphorylation and the reduction of FoxO1 in the nucleus led us to examine the binding of FoxO1 to the endogenous *Sox2* gene before and after treatment of i-OSKM-ESC with Dox. For this purpose, we conducted chromatin immunoprecipitation (ChIP) using two different FoxO1 antibodies (one recognizes the N-terminus of FoxO1, and the second recognizes the C-terminus), as well as a control IgG antibody used for normalization. Immunoprecipitated DNA was analyzed in triplicate by qPCR using primers specific to three different regions of the mouse *Sox2* gene. Two primer pairs were designed to examine FoxO1 binding to regions of the mouse *Sox2* gene, which map to regions in the human Sox2 gene reported recently to bind FOXO1 [19]. In our study, these regions are referred to as FoxO1 binding element (FBE)1 and 2 (Figure 7B). A third primer set was designed to amplify a region ∼4-kb downstream of the transcription start-site of the mouse *Sox2* gene, which was not expected to bind FoxO1 and, thus, served as a control region. After normalizing to the input samples and the control antibody, the fold-change of FoxO1 binding was determined by comparing FoxO1 antibody immunoprecipitation of FBE1 and FBE2 from untreated and Dox-treated i-OSKM-ESC sheared DNA with the control region of *Sox2*. ChIP analysis with both FoxO1 antibodies indicated that there was a statistically significant reduction (>60%) in FoxO1 binding at each FBE region of the *Sox2* gene after Dox treatment of i-OSKM-ESC (Figure 7C). Thus, our findings argue that the increases in pFoxO1(S253) (Figure 7A) contribute to decreases in *Sox2* expression at the RNA level when i-OSKM-ESC are treated with Dox (Figure 6A).

## Discussion

Previous studies focused heavily on the transcriptional circuitry of ESC [1]. More recently, studies have examined how signaling networks in ESC influence self-renewal and pluripotency of ESC by regulating critical transcription factors [10], [12], [18], [39], [40]. However, significant gaps remain in understanding how signaling networks and the transcriptional circuitry are integrated in ESC. Moreover, although the complex regulation of key transcription factors by signaling networks have received considerable attention [21], the effects of key transcription factors on the regulation of signaling networks has received far less attention. The findings reported in this study demonstrate that elevating Sox2 in conjunction with Oct4, Klf4 and c-Myc increases the phosphorylation of AKT and activates a negative feedback loop that helps to tightly regulate the levels of Sox2 in ESC. Interestingly, although endogenous Sox2 decreased following treatment of i-OSKM-ESC with Dox, we did not observe reductions in the expression of endogenous Oct4 or Klf4, suggesting that the negative feedback loop in this system is specific to Sox2. In the case of Oct4, this was unexpected since previous studies observed decreases in Oct4 promoter activity when Oct4 levels are ectopically elevated [6], [7].

In this study, we observed the activation of a Sox2-negative feedback loop in two cellular models (i-Sox2-ESC, i-OSKM-ESC) where increases in Sox2 levels (∼2-fold) in ESC are driven from a Dox-inducible transgene [4], [23]. In i-OSKM-ESC, increases in AKT phosphorylation correlate with the elevation of Sox2 protein levels, followed by decreases in endogenous Sox2. Importantly, addition of AKT inhibitors to Dox-treated i-OSKM-ESC reversed the inhibition of the endogenous *Sox2* gene at both the protein and RNA levels. In this study, we also examined whether other inhibitors would reverse the inhibition of endogenous Sox2 when Dox is added to i-OSKM-ESC. GSK3-β, MEK and FGFR inhibitors were tested because they have been shown to maintain the self-renewal and pluripotency of ESC [13], and because GSK3-β and MEK have been shown to regulate critical transcription factors, including c-Myc and Nanog [10], [12], [39]. However, GSK3-β, MEK and FGFR inhibitors did not reverse the repression of the endogenous Sox2. Thus, AKT signaling plays a key role in limiting Sox2 levels in ESC, especially when Sox2 levels begin to rise above optimal levels.

Prior to our finding that inhibition of AKT signaling in Dox-treated i-OSKM-ESC leads to the re-expression of endogenous Sox2, one might have expected that inhibition of AKT would lead to a decrease in Sox2 levels, because other studies have shown that AKT directly phosphorylates Sox2, which increases Sox2 stability [18]. Importantly, our data reflects both a role of AKT in the regulation of Sox2 protein stability and a role in regulating *Sox2* gene expression at the RNA level. Decreases in exogenous Sox2 protein after treatment with AKT inhibitors in the presence of Dox (Figures 3) supports the finding that AKT plays a role in Sox2 protein stability [18]; whereas, an increase in endogenous Sox2 after AKT inhibition in the presence of Dox (Figures 2, 3 and 5) argues that AKT influences *Sox2* expression through a novel mechanism at the mRNA level. In this regard, we tested whether endogenous *Sox2* gene expression was regulated by AKT signaling using RT-qPCR analysis of the 3'UTR of *Sox2*. We observed a small increase in endogenous *Sox2* mRNA when i-OSKM-ESC were treated with an AKT inhibitor, and a decrease of more than 50% in endogenous *Sox2* mRNA with Dox treatment, which was reversed by AKT inhibition in Dox-treated i-OSKM-ESC (Figure 6A). The decreases in *Sox2* mRNA in Dox-treated i-OSKM-ESC do not appear to be due to increases in the degradation of *Sox2* mRNA, because we determined that expression of miRNAs previously shown to target *Sox2* mRNA did not increase. In fact, the levels of three miRNAs known to regulate *Sox2* mRNA decreased in Dox-treated i-OSKM-ESC. Furthermore, there was not change in *Sox2* mRNA stability when Dox was added. Collectively, our findings argue that signaling through AKT protects Sox2 protein from degradation, while also inhibiting *Sox2* transcription. Conversely, our studies suggest that if AKT signaling decreases, thereby making Sox2 protein more vulnerable to protein degradation, *Sox2* transcription would increase to compensate for the decrease in Sox2 protein. Thus, the restoration of endogenous Sox2 expression at the protein level when AKT inhibitors are added to the Dox-treated i-OSKM-ESC appears to be the result of a delicate balance between the increase in *Sox2* at the RNA level and the decrease in Sox2 protein stability. This dual mechanism further highlights the importance of AKT signaling in promoting the self-renewal and pluripotency of ESC, which requires tightly regulated Sox2 levels [4]. Interestingly, the potential role of AKT, if any, on the stability of *Sox2* mRNA will require further study. In this regard, we did not observe a change in *Sox2* mRNA stability when Dox was added to i-OSKM-ESC. However, we observed a small increase in *Sox2* mRNA stability when AKTiV was added to Dox-treated i-OSKM-ESC. Thus, if AKT influences *Sox2* mRNA stability, even to a small extent, it would only increase the complexity of the roles played by AKT in the fine-tuning of Sox2 levels in ESC.

Our findings identify AKT as a significant player in *Sox2* mRNA regulation. To determine how AKT limits Sox2 expression, we examined the influence of AKT on three of its known downstream targets (GSK3-β, S6K, and FoxO1). Although phosphorylation of GSK3-β and S6K increased following elevation of Sox2 in Dox-treated i-OSKM-ESC, inhibition of neither GSK3-β (Figure 2A) nor S6K (Figure 5B) induced re-expression of endogenous Sox2, which is observed when AKT is inhibited. However, we also observed an increase of pFoxO1(S253) in the cytoplasm of Dox-treated i-OSKM-ESC, which was blocked by AKT inhibition (Figure 7A). Additionally, the total levels of FoxO1 decreased in the nucleus of Dox-treated i-OSKM-ESC and increased in the cytoplasm, which is consistent with earlier reports that the increased phosphorylation of FoxO1 leads to a shift in its subcellular localization [20]. Furthermore, FoxO1 is essential for the pluripotency of ESC, and the unphosphorylated form of FoxO1 binds the promoter of the human *SOX2* gene [19]. Thus, the increase in pFoxO1(S253) and translocation to the cytoplasm is consistent with our finding that endogenous *Sox2* mRNA decreases when AKT phosphorylation increases.

**Figure 8:**
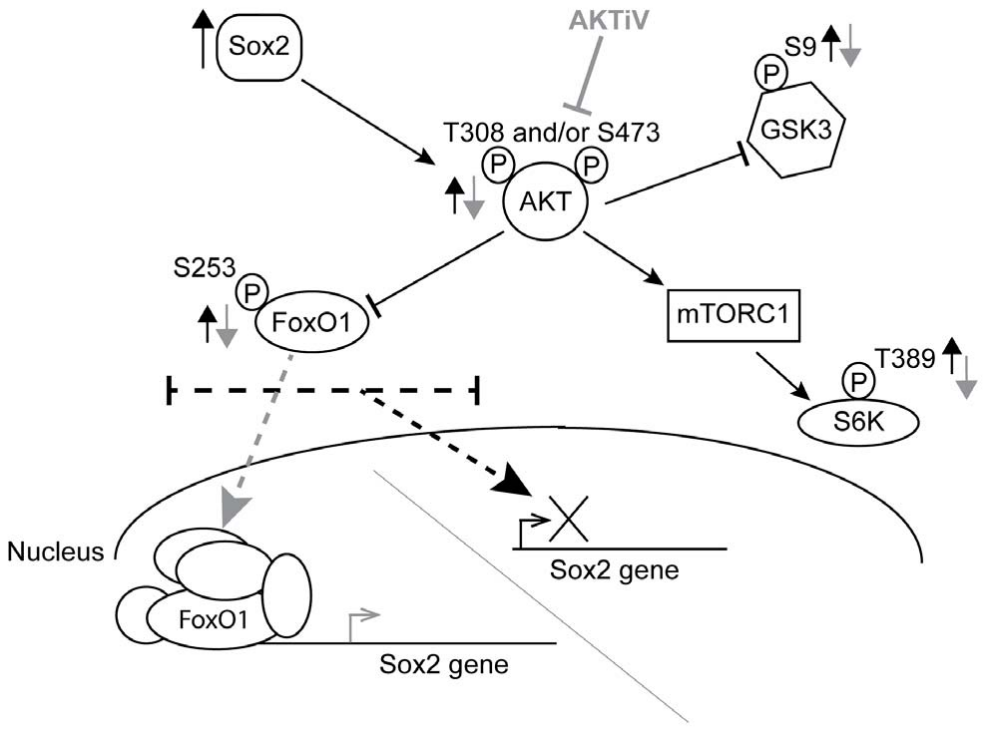
An AKT-mediated negative feedback loop tightly controls *Sox2* gene expression upon elevation of Sox2 protein. The effects of elevating Sox2 in ESC are represented by black arrows, and the effects of treating ESC with AKTiV are represented by gray arrows.

In further support of our contention that increased phosphorylation of FoxO1 in Dox-treated i-OSKM-ESC leads to decreases in *Sox2* transcription, we determined that FoxO1 binding at both *Sox2* FBE sites decreased by more than 60% in the presence of Dox (Figure 7C). Collectively, our findings support the following model. Elevated levels of Sox2 activate a Sox2-negative feedback loop that leads to increases in AKT activation, which, in turn, leads to increased pFoxO1(S253) that is sequestered in the cytoplasm and unable to bind the *Sox2* gene. As a result, transcription of *Sox2* decreases (Figure 8). Together, our findings argue that AKT modulation of FoxO1 binding to the *Sox2* gene plays an important role in the *Sox2*-negative feedback loop in ESC.

The findings reported in this study have at least two important implications beyond the regulation of *Sox2* by AKT in ESC. Several studies have shown that Sox2 levels influence the frequency of reprogramming. When the levels of Sox2 are too high, the frequency of reprogramming decreases [41]–[43]. Other studies have shown that AKT-mediated phosphorylation of Sox2(T118) enhances the frequency of reprogramming [18], presumably due to increases in the stability of Sox2. Thus, manipulation of the level of AKT activity is likely to enhance reprogramming depending on when and how much Sox2 expression is required during reprogramming. However, our finding that *Sox2* mRNA expression is regulated by AKT suggests that the improvements in reprogramming efficiency by modulation of AKT activity could be a complex undertaking. Interestingly, other studies have reported that the epigenetic reprogramming can either be increased or decreased by forced activation of AKT depending on the method of nuclear reprogramming [44]. Hence, we suspect that cells undergoing reprogramming may become more sensitive to the modulation of AKT signaling when the endogenous *Sox2* gene is activated, due to dual regulation of Sox2 stability and *Sox2* transcription by AKT.

In addition to reprogramming, our findings provide an important example of the mechanistic integration between the transcription circuitry in ESC and the signaling networks known to control the fate of ESC. While other investigators have reported the important influence of signaling on other critical ESC factors, such as c-Myc, Nanog, and Klf4 [10], [12], [39], [40], our findings provide new and important insights into the complex regulation of Sox2 in ESC by a negative feedback loop where increases in Sox2 activate AKT, which then leads to decreases in the expression of endogenous *Sox2* at the RNA level. Hence, our findings, and the work of Jeong et al. [18] regarding AKT regulation of Sox2 stability, demonstrate how AKT signaling provides a means of sensitive control over Sox2 expression in ESC.

In conclusion, the work described in this study investigated the poorly understood role of signaling pathways in the regulation of Sox2 expression when the levels of Sox2 rise above optimal levels in ESC. We determined that elevation of exogenous Sox2 (upon treatment with Dox) leads to increases in the activity of AKT and decreases in endogenous Sox2 expression. Importantly, inhibition of AKT in the presence of Dox leads to a re-expression of endogenous *Sox2* at both the RNA and the protein level. Equally important, we demonstrate that increased activation of AKT in Dox-treated i-OSKM-ESC leads to increases in phosphorylation of FoxO1, as well as decreases in FoxO1 binding to both FBE sites within the upstream regulatory regions of *Sox2*. Overall, our studies suggest that increases in the activity of AKT lead to decreases in the transcription of the endogenous *Sox2* gene, at least in part, due to the loss of nuclear FoxO1. These studies not only illustrate the tight integration of the signaling networks and transcriptional circuitry in ESC, they also emphasize the importance of understanding negative feedback loops that control the self-renewal of pluripotent stem cells and which are likely to play important roles during development and cellular reprogramming.

## Supporting Information

Figure S1 **Signaling upstream of PI3K/AKT and endogenous Sox2 expression.** i-OSKM-ESC were seeded at 1.5×10^6^ per 100 mm dish with or without 4 μg/ml Dox for 24 hours. (A) After the initial 24 hours, the cells were refed with fresh medium with or without 4 μg/ml Dox, and treated with 100 nM FGFRi (A), 50 nM Srci (B), or 1 µM PTENi (C) for an additional 24 hours where indicated. 48 hours after the cells were plated, nuclear and cytoplasmic protein extracts were prepared and equal amounts of nuclear and cytoplasmic protein were loaded into each well of an SDS-PAGE. Western blot analysis was performed by probing for Sox2 and HDAC1. HDAC1 was used as a loading control. (D) i-OSKM-ESC were seeded at 1.5×10^6^ per 100 mm dish. After 24 hours, cells were refed with fresh media with or without 4 μg/ml Dox for 48 hours. Whole cell protein extracts were prepared and equal amounts of protein were loaded into each well of an SDS-PAGE. Western blot analysis was performed by probing for ERas, Sox2, and HDAC1. HDAC1 was used as a loading control.
(TIF)

Table S1 Inhibitors used.
(DOC)

Table S2 Antibodies for western blot analyses.
(DOC)

Table S3 ChIP Analysis qPCR Primer Information
(DOC)
