# Supplementary figures and images for: Sox2 Expression Is Regulated by a Negative Feedback Loop in Embryonic Stem Cells That Involves AKT Signaling and FoxO1
Source: PLoS One. 2013 Oct 8;8(10):e76345. doi: 10.1371/journal.pone.0076345 (PMC3792943; doi:10.1371/journal.pone.0076345)

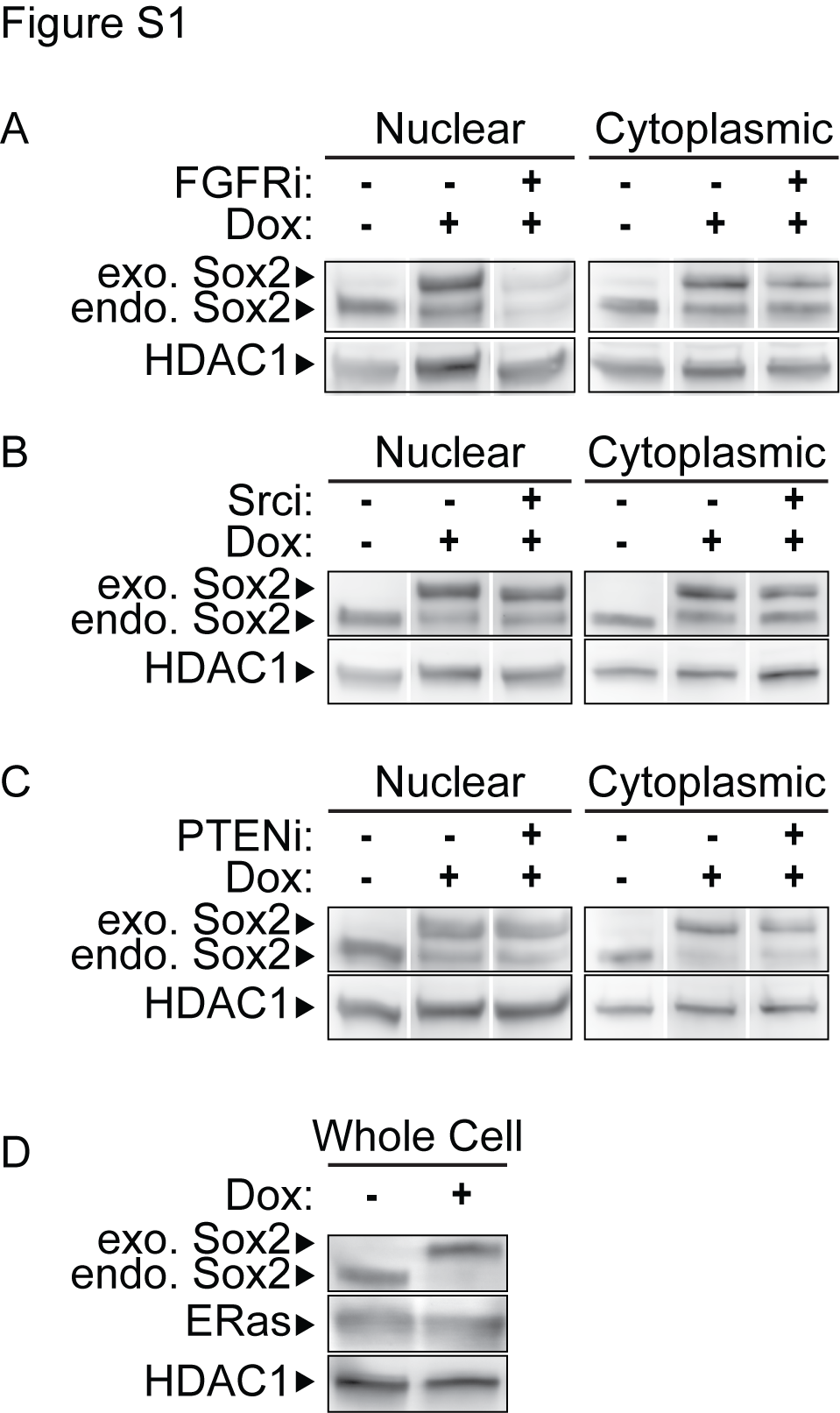

Supplement: Figure S1 — Signaling upstream of PI3K/AKT and endogenous Sox2 expression. i-OSKM-ESC were seeded at 1.5×10^6^ per 100 mm dish with or without 4 μg/ml Dox for 24 hours. (A) After the initial 24 hours, the cells were refed with fresh medium with or without 4 μg/ml Dox, and treated with 100 nM FGFRi (A), 50 nM Srci (B), or 1 µM PTENi (C) for an additional 24 hours where indicated. 48 hours after the cells were plated, nuclear and cytoplasmic protein extracts were prepared and equal amounts of nuclear and cytoplasmic protein were loaded into each well of an SDS-PAGE. Western blot analysis was performed by probing for Sox2 and HDAC1. HDAC1 was used as a loading control. (D) i-OSKM-ESC were seeded at 1.5×10^6^ per 100 mm dish. After 24 hours, cells were refed with fresh media with or without 4 μg/ml Dox for 48 hours. Whole cell protein extracts were prepared and equal amounts of protein were loaded into each well of an SDS-PAGE. Western blot analysis was performed by probing for ERas, Sox2, and HDAC1. HDAC1 was used as a loading control. (TIF) [file pone.0076345.s001.tif]
