# Supplementary material for: Sox2 Expression Is Regulated by a Negative Feedback Loop in Embryonic Stem Cells That Involves AKT Signaling and FoxO1
Source: PLoS One. 2013 Oct 8;8(10):e76345. doi: 10.1371/journal.pone.0076345 (PMC3792943; doi:10.1371/journal.pone.0076345)
Supplement: Table S1 — Inhibitors used. (DOC) [file pone.0076345.s002.doc]

**Table S1. Inhibitors used**

| **Inhibitor Name** | **Abbreviation** | **Amount** | **Company, Location** |
| --- | --- | --- | --- |
| AKT inhibitor, Triciribine | AKTiV | 5 μM | Calbiochem, EMD Millipore, Billerica, MA |
| AKT1/2 kinase inhibitor A6730 | AKT1/2i | 5 μM | Sigma-Aldrich, St. Louis, MO |
| GSK3-β inhibitor, CHIR99021 | CHIR | 3 μM | Selleck, Houston, TX |
| MEK inhibitor, PD0325901 | MEKi | 0.4 μM | Selleck, Houston, TX |
| PI3K inhibitor, LY294002 | LY | 10 μM | Selleck, Houston, TX |
| PI3K inhibitor, Wortmannin | WT | 200nM | Calbiochem, EMD Millipore, Billerica, MA |
| S6K inhibitor, PF-4708671 |  | 6 μM | Sigma-Aldrich, St. Louis, MO |
| FGFR inhibitor, PD173074 | FGFRi | 100 nM | Selleck, Houston, TX |
| Src inhibitor, S1021 Dasatinib | Srci | 50 nM | Selleck, Houston, TX |
| PTEN inhibitor, SF1670 | PTENi | 1 µM | Cellagen Technology, San Diego, CA |
