# Supplementary material for: Sox2 Expression Is Regulated by a Negative Feedback Loop in Embryonic Stem Cells That Involves AKT Signaling and FoxO1
Source: PLoS One. 2013 Oct 8;8(10):e76345. doi: 10.1371/journal.pone.0076345 (PMC3792943; doi:10.1371/journal.pone.0076345)
Supplement: Table S2 — Antibodies for western blot analyses. (DOC) [file pone.0076345.s003.doc]

**Table S2. Antibodies for western blot analyses**

| **Primary Antibody** | **Dilution** | **Company** |
| --- | --- | --- |
| Sox2 (#2683) | 1:5000 | Epitomics |
| HDAC1 (ab7028) | 1:5000 | Abcam |
| pAKT(T308) (#2965) | 1:1000 | Cell Signaling |
| pAKT(S473) (#4058) | 1:1000 | Cell Signaling |
| AKT (#9272) | 1:1000 | Cell Signaling |
| pGSK3-β(S9) (#9336) | 1:1000 | Cell Signaling |
| FoxO1 (#2880) | 1:1000 | Cell Signaling |
| pFoxO1(S256) (#9461) | 1:500 | Cell Signaling |
| pS6K(T389) (#9205) | 1:500 | Cell Signaling |
| c-Myc (#06-340) | 1:500 | Millipore |
| ERas (A-18) (sc-51072) | 1:500 | Santa Cruz |
|  |  |  |
| **Secondary Antibodies** | **Dilution** | **Company** |
| Anti-rabbit AP conjugate (A3687) | 1:10000 | Sigma-Aldrich |
| Anti-mouse AP conjugate (A4312) | 1:10000 | Sigma-Aldrich |
| Anti-goat AP conjugate (A4187) | 1:10000 | Sigma-Aldrich |
