# Supplementary material for: Sox2 Expression Is Regulated by a Negative Feedback Loop in Embryonic Stem Cells That Involves AKT Signaling and FoxO1
Source: PLoS One. 2013 Oct 8;8(10):e76345. doi: 10.1371/journal.pone.0076345 (PMC3792943; doi:10.1371/journal.pone.0076345)
Supplement: Table S3 — ChIP Analysis qPCR Primer Information (DOC) [file pone.0076345.s004.doc]

**Table S3. ChIP Analysis qPCR Primer Infomation**

| **Primer Name** | **Primer Sequences (mouse)** | **Trendline Equation** | **Primer Efficiency Equation** |
| --- | --- | --- | --- |
| Sox2 H/P | Forward 5’-CATTTATTCAGTTCCCAGTCC-3’  Reverse 5’-CCGTCATTTGGGTCTTTATTC-3’ | y = -3.633x + 36.899 | =10^((Ct Value - 36.899)/-3.633) |
| FBE 1^*^ | Forward 5’-CATCACATGGATGGTTGTCTATTAACTTGTTCA-3’  Reverse 5’-  CTCCCAGCCCTAGTCTTAAAGAGGCAGC-3’ | y = -1.842x + 41.080 | =10^((Ct Value - 41.08)/-1.842) |
| FBE 2^*^ | Forward 5’-CTTTGTTTGACTCCGTGTAGCGACA-3’  Reverse 5’-ATCTCCCATTGTCCAGACGTAAAG-3’ | y = -1.875x + 42.902 | =10^((Ct Value - 42.902)/-1.875) |

*Designed based off of primers used previously to recognize human FoxO1 binding regions within human Sox2 gene regulatory regions (Zhang et al., 2011).
